# Freezing of Gait in Parkinson’s Disease: An Overload Problem?

**DOI:** 10.1371/journal.pone.0144986

**Published:** 2015-12-17

**Authors:** Eric N. Beck, Kaylena A. Ehgoetz Martens, Quincy J. Almeida

**Affiliations:** 1 Sun Life Movement Disorders Research & Rehabilitation Centre, Wilfrid Laurier University, Waterloo, Ontario, Canada; 2 Department of Kinesiology and Physical Education, Wilfrid Laurier University, Waterloo, Ontario, Canada; 3 Department of Psychology, University of Waterloo, Waterloo, Ontario, Canada; University of California, Merced, UNITED STATES

## Abstract

Freezing of gait (FOG) is arguably the most severe symptom associated with Parkinson’s disease (PD), and often occurs while performing dual tasks or approaching narrowed and cluttered spaces. While it is well known that visual cues alleviate FOG, it is not clear if this effect may be the result of cognitive or sensorimotor mechanisms. Nevertheless, the role of vision may be a critical link that might allow us to disentangle this question. Gaze behaviour has yet to be carefully investigated while freezers approach narrow spaces, thus the overall objective of this study was to explore the interaction between cognitive and sensory-perceptual influences on FOG. In experiment #1, if cognitive load is the underlying factor leading to FOG, then one might expect that a dual-task would elicit FOG episodes even in the presence of visual cues, since the load on attention would interfere with utilization of visual cues. Alternatively, if visual cues alleviate gait despite performance of a dual-task, then it may be more probable that sensory mechanisms are at play. In compliment to this, the aim of experiment#2 was to further challenge the sensory systems, by removing vision of the lower-limbs and thereby forcing participants to rely on other forms of sensory feedback rather than vision while walking toward the narrow space. Spatiotemporal aspects of gait, percentage of gaze fixation frequency and duration, as well as skin conductance levels were measured in freezers and non-freezers across both experiments. Results from experiment#1 indicated that although freezers and non-freezers both walked with worse gait while performing the dual-task, in freezers, gait was relieved by visual cues regardless of whether the cognitive demands of the dual-task were present. At baseline and while dual-tasking, freezers demonstrated a gaze behaviour that neglected the doorway and instead focused primarily on the pathway, a strategy that non-freezers adopted only when performing the dual-task. Interestingly, with the combination of visual cues and dual-task, freezers increased the frequency and duration of fixations toward the doorway, compared to non-freezers. These results suggest that although increasing demand on attention does significantly deteriorate gait in freezers, an increase in cognitive demand is not exclusively responsible for freezing (since visual cues were able to overcome any interference elicited by the dual-task). When vision of the lower limbs was removed in experiment#2, only the freezers’ gait was affected. However, when visual cues were present, freezers’ gait improved regardless of the dual-task. This gait behaviour was accompanied by greater amount of time spent looking at the visual cues irrespective of the dual-task. Since removing vision of the lower-limbs hindered gait even under low attentional demand, restricted sensory feedback may be an important factor to the mechanisms underlying FOG.

## Introduction

Freezing of gait (FOG) is the manifestation of unintended halting of step-to-step production, and is perhaps the most severe motor symptom in Parkinson’s disease (PD). This faulty motor output from the basal ganglia is not fully understood, and is an unpredictable phenomenon that is difficult to elicit in an experimental setting [1]. As a result, spatiotemporal gait parameters have been used as a reliable predictor of freezing, for example, decreased step length and step time, decreased velocity, and increased step length and step time variability [1–5]. Multiple hypotheses have attempted to explain the underlying mechanism of freezing [6], however, a model that universally explains the occurrence of FOG has not yet been established.

Cognitive models of FOG hypothesize that diversion of attention away from walking (which is less automatically controlled in PD), is responsible for an interruption in motor output resulting in a freezing episode. Thus, when PD patients who experience freezing, (also known as ‘freezers’), perform a secondary task while walking, they demonstrate increased gait variability and a greater number of FOG episodes [7–12]. Thus, it seems that when gait is consciously controlled, attention can play a critical role in causing freezing episodes. However, in some cases directing attention toward walking does not alleviate freezing. For example, a recent study used laser visual cues to focus attention during walking, yet these cues were unable to improve gait in freezers [13]. It is also important to consider why freezing still occurs in conditions where very low cognitive demand is involved. One example of a simplistic task with low cognitive demand is approaching doorways, which have been found to increase step-to-step variability and number of freezing episodes [5,14–16]. While a cognitive explanation cannot be entirely ruled out, these research groups have suggested that FOG may be the result of an online sensory-perceptual impairment. More specifically, it has been hypothesized that freezers have an extreme dependency on vision, which is necessary to augment faulty proprioceptive feedback ascending from the lower limbs. If this were the case, then vision would be necessary to compensate for proprioceptive deficits, and visual cues would help identify step amplitude deficits that might otherwise go undetected by online proprioceptive processing. Based on this viewpoint, when freezers utilize vision to assess their proximity to an upcoming doorway (or obstacle), the perceptual distance judgment must be integrated with faulty online proprioception, thus leading to a mismatch and the necessity of a freeze to recalibrate the two forms of sensory feedback before continuing movement. In support of this, recent research has shown that when visual information about the body is removed, freezers experienced more freezing episodes compared to walking with their body illuminated [15]. Together, these types of studies [15,17–19] support a link between sensory feedback and FOG, suggesting that in the absence of vision of the lower limbs something about the online planning process must be interrupted.

Both the cognitive and sensory-perceptual viewpoints have merit, and offer their own perspectives as to why visual cues might be a helpful strategy to overcome FOG [13,20–22]. Those who support the cognitive model might argue that visual cues direct attention toward the planning of each discrete step [23,24]. However, visual cues have also been argued to compensate for faulty proprioceptive processing in PD [25–27] by providing visual verification of each step, more in support of a sensory-perceptual explanation [13]. To date, there has not been a direct comparison of these two important hypotheses to help us understand the underlying mechanisms of FOG. To do so, this study examined gait behavior in response to increased attentional and sensory-perceptual demand in isolation, and then in combination. By providing visual cues to walk on, the degree of gait interference associated with attentional and sensory-perceptual demand (level in which gait deteriorates as a result of dual-tasking or occluded vision of the lower limbs) can be determined, and provide insight as to which of these processes visual cues are aiding (and predominantly responsible for) FOG. Regardless, it seems clear that vision has a critical link to understanding attentional and sensory-perceptual influences on FOG.

One unique method of clarifying how vision is involved in planning movement (and potentially triggering FOG) is to evaluate gaze behaviors in freezers and non-freezers. If an attentional or sensory-perceptual impairment is responsible for the mechanism underlying FOG, then increasing the demand on these resources separately and in combination might show a very different pattern of gaze behaviours compared to non-freezers. Based on previous literature examining gaze behaviour in attentional deficits [28], it was expected that in a narrowed doorway, without a secondary-task to perform, freezers would demonstrate an increased frequency of fixations to the task relevant information (i.e. the doorway). Therefore, greater number of fixations might suggest a heightened attempt to adequately plan for the upcoming movement. If attention was divided (dual-task), it could be expected that the number of fixations would be even greater since fewer resources would be available to monitor relevant information about the task and the environment, and thus more fixations might be needed to adequately plan for the upcoming movement. However, if visual cues were provided to promote a strategy that directs attention to walking, greater frequency and duration of fixations would be expected towards the pathway, and less toward the door. If visual cues were provided while performing a dual-task, it was expected that freezers would fixate less on the pathway and more frequently on the doorway compared to the condition that included only visual cues since fewer available resources would limit their ability to adequately plan for the upcoming environment. It was also expected that these patterns of gaze behaviours would be similar between freezers and non-freezers, however those individuals who experience freezing would demonstrate a greater number of fixations for a shorter duration. It has also been well established that higher levels of tonic electrodermal activity (i.e. skin conductance levels) may indicate increased attention and vigilance [29–31]. Therefore, by combining skin conductance levels (SCL) and gaze measures, we attempted to understand which conditions were more attentionally demanding in addition to where individuals might be attending to. It was expected that both the dual-task and visual cues would lead to greater SCL in all participants compared to baseline.

On the other hand, if FOG is the result of a sensory-perceptual deficit, it might be expected that freezers would compensate for impaired proprioceptive processing with vision, by using visual cues to enhance walking performance. Therefore, while walking towards the narrow-doorway (without a dual-task or visual cues), it was expected that gaze would be directed toward the pathway providing relevant information about movement speed, and current/ future steps. Since dividing attention (dual-task) should not influence an existing sensory-perceptual deficit, freezers were expected to continue looking towards the pathway, similar to walking towards the narrow-doorway without a dual-task. Furthermore, the addition of visual cues might offer a target for freezers, providing visual verification of footfalls to each line, and more salient optic flow to compensate for a sensory-perceptual deficit. Thus, with visual cues, it was expected that freezers would fixate more often and for greater duration towards the pathway. With the addition of a dual-task, gaze behaviour towards the visual cues was not expected to change drastically, unless an overload of attention (to both dual-task and use visual cues) led to further gait impairment.

Since gaze behaviour would not allow inferences about what information is necessary to compensate for sensory-perceptual deficits, these dual-task and visual cues conditions were followed-up by removing vision of the lower-limbs to address this drawback. Previously we hypothesized that increased vision towards the pathway would be employed by freezers to compensate for a sensory-perceptual impairment (gait relevant information), thus we evaluated the extent to which deficits to gait result when removing vision of the lower-limbs.

The overall objective of this study was to disentangle the underlying mechanism of FOG and explore the interaction between cognitive and sensory-perceptual influences. In order to do so, two experiments were conducted where patients walked towards and through a narrow doorway. The first experiment aimed to investigate (i) whether diverting attention with a dual-task influences FOG and (ii) whether visual cues can improve freezing when attention is diverted with a dual-task. It was expected that diverting attention with a dual-task would lead to increased gait impairment (greater number of freezing episodes, decreased step length, and increased step-to-step variability). If visual cues act as an attentional strategy, then diverting attention with a dual-task might be expected to reduce the effectiveness of visual cues at improving gait and freezing, or elicit greater error in performance of the dual-task. However, if visual cues act as a sensory strategy (regardless of attention), then diverting attention with a dual-task would not interfere with gait improvements.

The second experiment aimed to investigate (i) whether sensory deficits influence FOG, and (ii) whether visual cues can improve gait impairments when sensory feedback is restricted. It was expected that restricting sensory feedback (removing vision of lower-limbs) would exacerbate gait impairment. If visual cues compensate for proprioceptive deficits by visually guiding step placements, then removing vision of the lower-limbs might be expected to reduce the effectiveness of visual cues at improving gait. If visual cues act as an attentional strategy, then removing vision of the lower-limbs would not interfere with attention, thus visual cues would remain effective. In order to fully understand the FOG mechanism that visual cues act on, the interaction between dual-task, restriction of sensory feedback, and visual cues was examined in the second experiment.

## General Methods

### Participants

This study involved 40 age-matched participants with PD, 20 of which were determined to experience FOG by a movement disorders specialist and 20 of which did not. All participants were recruited from the patient database at the Movement Disorders Research and Rehabilitation Centre (MDRC) at Wilfrid Laurier University in Waterloo, Canada. Both groups participated in two experiments. Conditions of each experiment were combined into one data collection period, with all trials randomized. Due to the length of the protocol, five freezers were able to complete all conditions from the first experiment and one condition from the second experiment as a result of the randomization of conditions. Exclusion criteria included individuals with a neurological disease other than PD, peripheral neuropathy, diabetes, visual impairments that were not corrected or disallowed individuals to see a doorway, unable to walk unassisted for 10m, or clinically diagnosed with dementia (as stated in the patient’s information chart from the patient database at the MDRC). Although less frequent than in the OFF state, FOG has been observed to occur when individuals are in the ON state of dopaminergic medication. To ensure greater ecological validity, and since the primary research question was to investigate the interaction between cognitive and sensory systems (which influence impaired movement in PD despite dopaminergic medication) all testing took place one hour after taking anti-Parkinsonian medication. All participants were informed of the requirements and experimental protocols of this study. Prior to testing, written consent was obtained according to the Declaration of Helsinki. The Research Ethics Board at Wilfrid Laurier University granted full ethical approval of this research study.

### Experimental Setup

**Fig 1:**
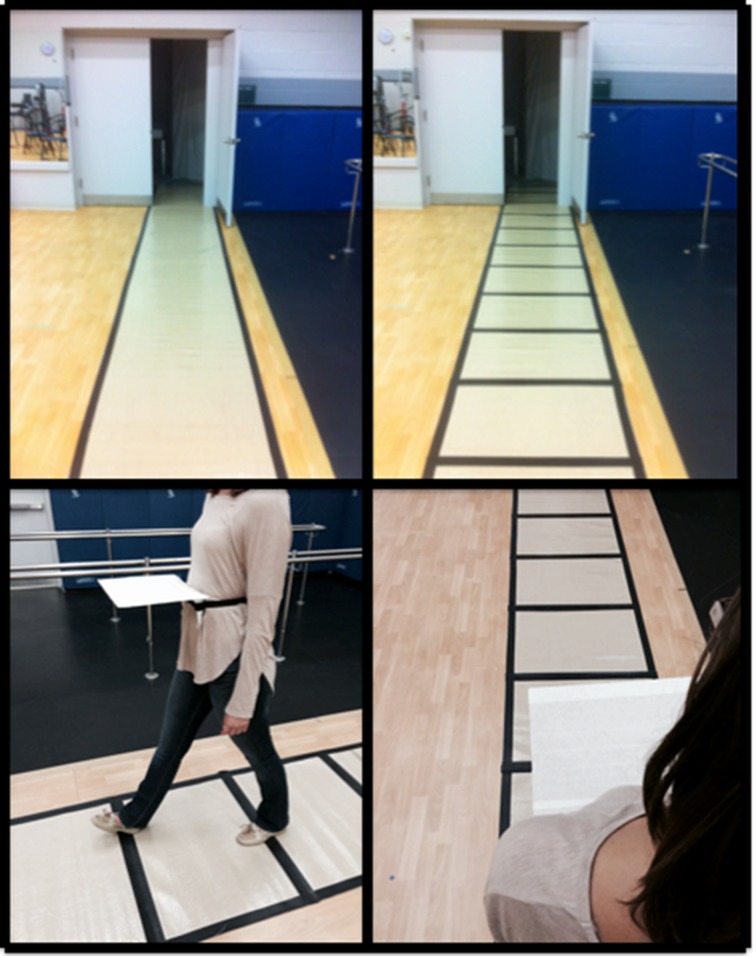
Experimental paradigms: No visual cues pathway (top left), visual cues pathway (top right) and device to occlude vision of the lower-limbs (bottom images).

Data collection took place in the rehabilitation studio of the Movement Disorders Research and Rehabilitation Centre. Since previous reports have demonstrated difficulty in eliciting FOG episodes, potentially as a result of heightened arousal from experimental paradigms [1,5], all trials involved walking toward and through an open and empty room with a narrowed doorway (a 0.225m wooden plank was added to the side of a 0.9m wide x 2.1m high doorframe creating a narrow doorway that was 0.675m wide x 2.1m high). All gait data was collected on a 7.92m long and 0.61m wide ProtoKinetics Movement Analysis Software™ electronic walkway carpet (Zeno Walkway–ProtoKinetics, Havertown, PA, USA) to capture spatiotemporal aspects of participant’s gait before the doorway. Two pathways of equal length (10m) that approached and continued beyond the narrowed doorway were utilized: (i) No visual cues (-VC)—a 10m long, 0.8m wide beige vinyl carpet that did not contain any lines was placed on top of the Zeno Walkway™ carpet; (ii) Visual cues (+VC)—a 10m long, 0.8m wide beige vinyl carpet that contained equidistantly 6cm wide black duct-tape striped lines positioned 65cm apart [32] was placed on top of the Zeno Walkway™ (Fig 1, top images). When visual cues were not present, participants were simply asked to walk toward and through the narrowed doorway at their preferred walking speed. Whereas, when visual cues were present, participants were instructed to walk towards and through the narrowed doorway while simultaneously stepping on each black line. Two additional manipulations were also utilized in the experimental protocols in combination with the visual cue manipulation. Firstly, a dual-task (+DT/-DT) was employed to investigate the influence of attention on FOG. Before any walking trials were performed, two seated baseline trials of dual-task performance were assessed. Participants were asked to listen to an audio track of random numbers greater than zero and less than 10. For each trial that included this task, the participants were assigned two specific digits and instructed to silently count (without the use of manual aid) the number of times those two digits were announced by the audio track, separately. The order in which digits were presented in the audio track was randomized differently for each trial. The auditory inter-stimulus interval was presented in a randomized manner, varying from 100ms to 1000ms, in order to prevent gait synchronization with the audio track. Participants were asked to continue to count the digits for the entire 12-second duration of the audio track. In trials where participants were asked to walk while simultaneously listening to the numbers, participants were asked to continue counting the digits regardless if they had finished the walking condition. At the end of the trial the participants were asked to inform the researcher of the number of times they heard the two digits. This was recorded and the difference between the participant’s response and the actual number of digits was calculated. Recording the errors helped distinguish if participants allocated attention to the digit-monitoring task or to walking. Secondly, a lower-limb occlusion manipulation (+LLO/-LLO, Fig 1, bottom images) was utilized to investigate the influence of sensory-perception on FOG. In this case, participants were asked to walk towards and through the narrowed doorway with a 25cm long, 30cm wide constructed cardboard device attached to the front of the participant’s waist by a Velcro® strap.

In all trials, spatiotemporal gait data was only collected prior to the doorway. Also, two spotters were present, one walking beside and slightly behind the participant along the pathway towards the narrowed doorway, and the second on the other side of the doorway, out of sight.

## Experiment 1: The Influence of Attention on Freezing of Gait

### Methods

To investigate the influence that attention has on FOG, participants completed three trials of two conditions on each of the two pathways. Each condition consisted of walking toward the narrow doorway (7.92m) and continuing into an empty room. Conditions were two levels of attentional load: (i) No dual-task (-DT)–walking through the narrow doorway and (ii) Dual-task (+DT)–walking through the narrow doorway while performing the digit-monitoring task, previously described, on either the (i) No visual cues pathway (-VC) or (ii) Visual cues pathway (+VC). In all cases, participants started each trial standing on the edge of the Zeno Walkway™ carpet. Participants were instructed to fixate on a yellow light-emitting diode placed directly beside the carpet and initiate walking at the moment they saw the light turn on.

### Data Analysis

#### Participant demographic analysis

In order to verify whether any significant differences between the freezers and non-freezers existed (with respect to age, sex, symptom severity, cognitive status, visual acuity, shoulder width, performance on parts A and B of the trail making test, and performance on the digit memory test), independent t-tests were utilized.

#### Gait data and statistical analysis

In the first experiment, data was statistically compared from two independent groups: 20 freezers and 20 non-freezers. The primary outcome measures utilized were FOG frequency and the average percent of trial spent frozen. FOG episodes were defined as a center of mass velocity that fell between 0m/s and one standard deviation of that individual’s average center of mass velocity [14,15]. Episodes were also confirmed subjectively through visual and video confirmation, and objectively through ProtoKinetics Movement Analysis Software™ estimate calculations of the center of mass velocity. The difficulties in eliciting FOG episodes in a laboratory setting have been well documented [1,5], and these difficulties were certainly present in the current study. Only four freezers experienced FOG episodes in the data collection area, and for this reason, statistical analyses of the FOG measures were not made (Table 1 demonstrates these two measures of FOG among the four participants that experienced freezing episodes in experiment one). However, as previously discussed, particular changes to walking patterns, not typical of gait, have been demonstrated as reliable predictors of FOG (i.e. step length variability and step time variability) [2,5]. For this reason, spatiotemporal parameters of gait were analyzed and compared between conditions and groups. Gait was only analyzed prior to the narrow doorway, even though individuals were instructed to continue walking passed that point to ensure that deceleration of walking did not influence spatiotemporal gait outcomes. To control for outliers and determine the influence of the conditions on walking, FOG episodes were removed from analysis, along with two steps before and after each FOG episode [2]. Gait variables measured in all conditions were: mean step length (cm), step length variability (CV), mean step time (sec.), step time variability (CV), double support time percentage (DST%), double support time percentage variability (CV), and gait velocity (cm/s). Both left and right footfalls were collapsed and results were analyzed using StatSoft STATISTICA 8.0.550.

**Table 1:** Number of freezing of gait episodes and average percentage of trials spent frozen with respect to the four individuals with Parkinson’s disease who experienced freezing of gait episodes in experiment#1.

Patient	Number of FOG Episodes				Average Percentage of Trial Spent Frozen (%)			
	No Dual-Task		Dual-Task		No Dual-Task		Dual-Task	
	*No VC*	*VC*	*No VC*	*VC*	*No VC*	*VC*	*No VC*	*VC*
1	0	0	3	2	0	0	56.7	35.1
2	3	0	7	5	54.8	0	65.6	48.0
3	0	0	0	2	0	0	0	12.0
4	0	0	0	1	0	0	0	17.1

FOG, freezing of gait; VC, visual cues; VC+LLO, visual cues and lower-limbs occlusion

In the first experiment, a four factor mixed repeated measures ANOVA (group x dual task x visual cues x trial) assessed group differences in each condition while walking on each pathway. Since disease severity measured by the Unified Parkinson’s disease Rating Scale-III (UPDRS-III) was different between groups (discussed further in the results), scores were included as a covariate variable on a secondary analysis (ANCOVA). Tukey’s Honest Significant Difference (HSD) post hoc was utilized to determine where significant differences were with respect to main effects and interactions.

#### Gaze data and statistical analysis

To investigate gaze behaviour that freezers and non-freezers adopted as they navigated the experimental paradigm, a wireless mobile eye tracker (Mobile Eye ASL- Applied Science Laboratories, Bedford, MA, USA) was utilized with a data collection sample frequency of 30 Hz. To calibrate the eye tracker system, a 9-point calibration protocol was used. Participants were asked to fixate their gaze on a three-by-three grid, beginning with the top-left point, and sequentially fixate on all nine grid-points. Gaze fixations were defined as a stabilization of eye gaze track (1.0 degree of deviation) on a specific location in the environment for a minimum duration of 99.99ms. Participant’s gaze data was excluded from analysis if the displayed eye track was missing for more than 25% of the travel time [33]. For this reason, calibration complications resulted in exclusion of some participants’ gaze data. In experiment #1 gaze data from 14 freezers and 17 non-freezers is reported on. To reveal modifications to gaze behaviour that is associated with planning for upcoming steps, each trial was analyzed in an early, middle, and late phase, and the number of footfalls in the trial were used as criteria for phase separation [18,19,34–36]. Gaze and gait data were temporally synchronized using the Zeno Walkway ProtoKinetics light-emitting diode that was triggered at the onset of gait data collection and visible by the scene camera of the eye tracker system. In the offline analysis, appearance of the light-emitting diode represented zero time, allowing for synchronization of gaze and gait data. The late phase was defined as the last four footfalls prior to the doorway (n-4, n-3, n-2, and n-1), which included all gaze data between the toe-off of the fifth step prior to the doorway and toe-off of the last step before the doorway. The middle phase included all gaze data between the toe-off of the ninth step prior to the doorway and the toe-off of the fifth step prior to the doorway (n-8, n-7, n-6, and n-5). The early phase included all footfalls that occurred prior to the toe-off of the ninth step before the doorway. In each phase, three areas of interest were included in the analysis: i) Pathway–the designated path of walking prior to the plane of the doorway (conditions that included visual cues were not analyzed differently than conditions absent of visual cues); ii) Doorway–the doorframe of the door and carpet area in the plane of the doorway; and iii) Through–all gaze fixations past the doorframe. Gaze data analysis was completed using Results Plus GM™ software (ASL- Applied Science Laboratories, Bedford, MA, USA). To normalize for walking velocity between conditions, pathways and groups, the percentage of fixations and percentage of total fixation duration on the areas of interest were the only variables analyzed. In the first experiment, a five factor mixed repeated measures ANOVA (group x dual-task x visual cues x phase x areas of interest) assessed group differences in each condition. Individual gaze data in each condition was averaged between three trials to provide variance to all dependent variables. To determine where significant differences were with respect to main effects and interactions, Tukey’s HSD post hoc test was utilized. However, to date, no study has investigated gaze behaviour in freezers while they navigate towards and through a doorway. Due to the novelty of this study and the intention to guide future research with the patterns of gaze behaviour investigated, a less conservative Fisher’s Least Significant Difference (LSD) post hoc test was also utilized [37] and reported clearly in the results section.

#### Skin Conductance data and statistical analysis

Electrodermal activity was collected with Q sensor cuffs (collection at a frequency of 8 Hz) strapped to the hypothenar eminence on each of the patients’ palms [31,38]. Q sensor data collection was synchronized with a button press by the researcher just prior to the light-emitting diode indicating the start of each trial and a second button press after participants walked through the doorway and completed the dual-task (if performed in that condition). The average SCL over the period of the entire trial (duration of time from first button press to the second button press) were used to quantify electrodermal activity throughout each walking trial. A four factor mixed repeated measures ANOVA (group x dual task x visual cues x trial) assessed group differences in each condition while walking on each pathway. Tukey’s HSD post hoc was utilized to determine where significant differences were with respect to main effects and interactions.

### Results

#### Participant demographics

No significant differences were found between groups with respect to age, shoulder width, visual distance acuity, or performance on the Montreal Cognitive Assessment. However, freezers (38.8) had significantly higher UPDRS-III scores than the non-freezers (24.7) (t(38) = 4.87, p<0.0001, Table 2).

**Table 2:** Demographic characteristics of participants with Parkinson’s disease who experience freezing of gait (freezers) and do not experience freezing of gait (non-freezers) in experiment#1.

Groups	AGE	Sex	UPDRS-III	MOCA	Distance Acuity	Shoulder Width (cm)	TMT A (sec)	TMT B (sec)	DMT Forward (score/16)	DMT Backward (score/14)
Freezers (n = 20)	72 (7.2)	2 F, 18 M	38.8* (10.6)	25.1 (3.4)	20/60	47 (4.0)	51.0 (14.8)	144.8* (64)	10.6 (2.3)	5.9 (1.5)
Non-Freezers (n = 20)	70.5 (8.4)	2 F, 18 M	24.7 (7.5)	26.5 (2.1)	20/70	48.5 (4.3)	41.9 (12.8)	85.8 (34)	10.4 (2.7)	6.9 (1.8)

n, number of participants; UPDRS-III, Unified Parkinson’s Disease Rating Scale-III; MOCA, Montreal Cognitive Assessment; TMT A and B, Trail Making Test Parts A and B; DMT, Digit Memory Test.

* represents a significant difference between groups at the p<0.05 level.

#### Gait parameters

**Fig 2:**
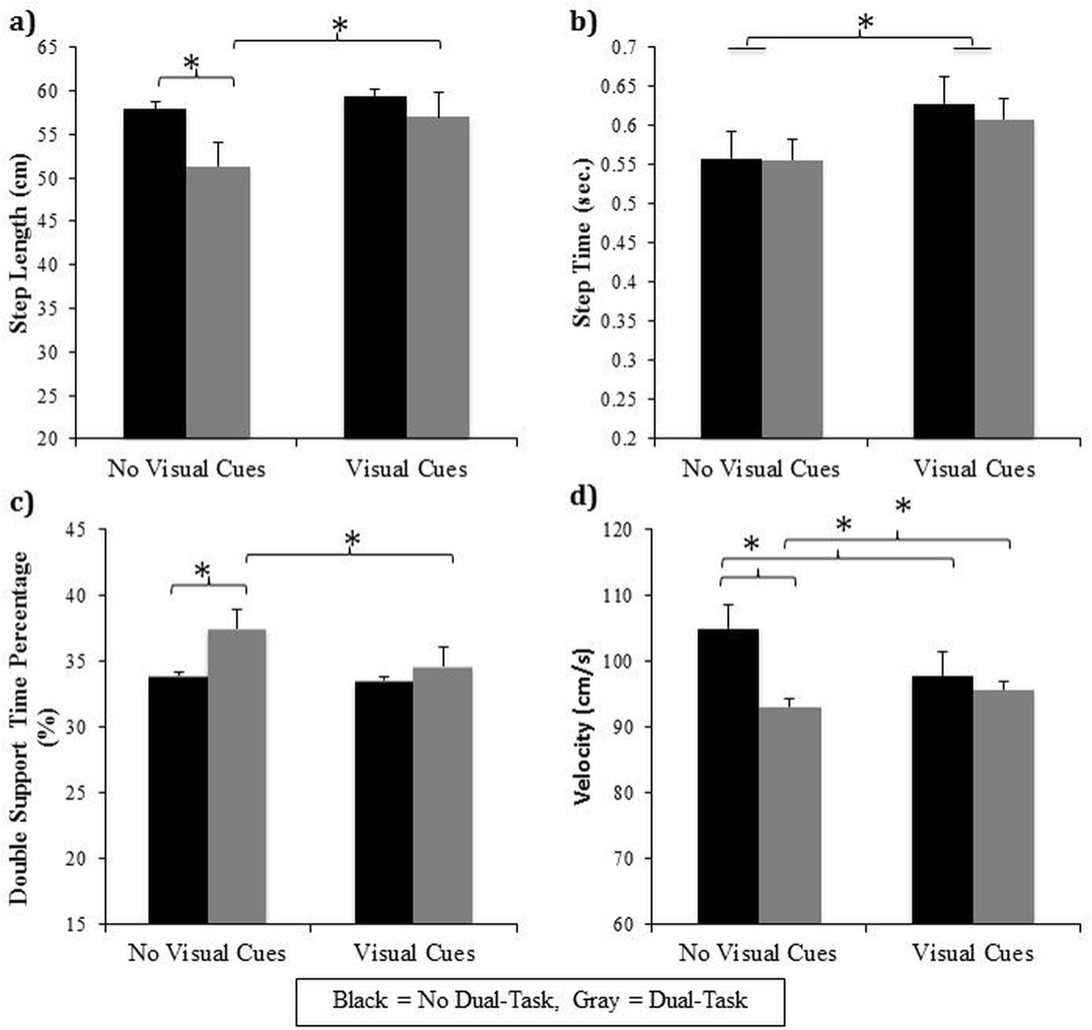
Significant gait interactions in Experiment #1 between dual-task and visual cues: Graphical illustration of a) step length, b) step time, c) double support time percentage, and d) velocity collapsed across groups when participants performed either the “dual-task” (gray) or “no dual-task” (black) conditions while walking on either the “no visual cues” pathway or “visual cues” pathway. * represents a significant difference at the p<0.05 level.

All significant and non-significant gait main effects and interactions are presented in Table 3. Significant interactions between dual-task and visual cues were found for step length (F(1,29) = 31.57, p<0.0001), step time (F(1,29) = 55.97, p<0.0001), DST% (F(1,29) = 19.62, p = 0.0001), and velocity (F(1,29) = 5.26, p = 0.029) (see Fig 2). Tukey’s post hoc analysis revealed multiple key differences. Firstly, both groups significantly decreased their step length (p<0.001), increased DST% (p<0.001) and walked significantly slower (p<0.001) when walking toward a door while performing a dual-task compared to walking without the dual-task (+DT/-VC vs. -DT/-VC). Secondly, both groups had significantly greater step time (p<0.001) and slower velocity (p<0.001) when visual cues were present compared to walking without visual cues available (-DT/+VC vs. -DT/-VC). However, step length (p = 0.624) and DST% (p = 0.488) were not significantly impacted by the presence of visual cues. Third, when the dual-task was performed in combination with visual cues (+DT/+VC), both groups significantly increased their step length (p<0.001), step time (p<0.001), and velocity (p = 0.017), while decreased their DST% (p<0.001) compared to the +DT/-VC condition. Finally, when visual cues were provided, step length, step time, DST% and velocity were similar regardless of the demands of a dual-task (–DT/+VC vs. +DT/+VC) indicating that when visual cues were present, the dual-task did not influence PD participants’ gait.

**Table 3:** Experiment #1 spatiotemporal gait parameter effects and interactions.

	Conditions				Effects (p values)																
	No Dual-Task		Dual-Task																		
	No Visual Cues	Visual Cues	No Visual Cues	Visual Cues		Group	DT	Group*DT	VC	Group* VC	Trial	Group* Trial	DT* VC	Group* DT*VC	DT* Trial	Group* DT*Trial	VC* Trial	Group* VC* Trial	DT* VC* Trials	Group* DT* VC*Trial	UPDRS
***Step Length (cm)***					ANOVA	0.0014	<0.0001	0.061	0.0007	0.0026	0.0091	0.337	<0.0001	0.717	0.538	0.256	0.0035	0.203	0.623	0.883	
					ANCOVA	0.128	0.214	0.272	0.187	0.0103	0.385	0.318	0.364	0.578	0.251	0.713	0.54	0.828	0.951	0.952	0.081
Freezers	49.23 (13.25)	54.66 (15.12)	41.35 (14.04)	49.31 (18.55)																	
non-Freezers	65.58 (8.57)	63.46 (6.15)	61.04 (7.48)	64.42 (2.90)																	
***Step Length Variability (CV)***					ANOVA	0.0231	0.0003	0.0153	0.112	0.435	0.718	0.51	0.542	0.935	0.342	0.977	0.713	0.578	0.089	0.625	
					ANCOVA	0.384	0.3	0.061	0.194	0.234	0.33	0.971	0.245	0.459	0.043	0.192	0.224	0.852	0.657	0.62	0.132
Freezers	12.62 (9.86)	13.77 (15.69)	17.75 (13.40)	15.04 (13.66)																	
non-Freezers	6.00 (3.61)	5.47 (3.23)	6.32 (2.91)	5.48 (2.97)																	
***Step Time (sec.)***					ANOVA	0.08	0.92	0.661	<0.0001	0.0139	<0.0001	0.0352	0.0292	0.469	0.0019	0.804	<0.0001	0.103	<0.0001	0.547	
					ANCOVA	0.256	0.418	0.39	0.0441	0.0333	0.0001	0.034	0.343	0.417	0.246	0.777	0.0434	0.262	0.281	0.941	0.785
Freezers	0.57 (0.09)	0.68 (0.19)	0.56 (0.09)	0.64 (0.14)																	
non-Freezers	0.55 (0.05)	0.58 (0.06)	0.55 (0.05)	0.58 (0.06)																	
***Step Time Variability (CV)***					ANOVA	0.0122	0.988	0.17	0.0026	0.746	0.005	0.33	0.0016	0.848	0.0227	0.235	0.76	0.355	0.346	0.474	
					ANCOVA	0.154	0.885	0.356	0.527	0.889	0.922	0.782	0.272	0.738	0.587	0.313	0.825	0.664	0.446	0.851	0.424
Freezers	7.54 (8.31)	10.77 (7.28)	8.55 (5.51)	9.46 (5.77)																	
non-Freezers	4.49 (4.56)	5.74 (1.99)	4.89 (2.29)	5.42 (1.62)																	
***Double Support Time Percentage (%)***					ANOVA	0.326	<0.0001	0.582	0.0001	0.0299	0.0451	0.497	0.0001	0.76	0.077	0.2	0.128	0.628	0.735	0.969	
					ANCOVA	0.367	0.225	0.657	0.104	0.0432	0.285	0.214	0.887	0.393	0.21	0.134	0.516	0.838	0.772	0.828	0.0132
Freezers	36.65 (8.43)	35.46 (10.35)	41.22 (10.54)	37.72 (11.92)																	
non-Freezers	31.60 (4.81)	31.96 (4.66)	33.86 (4.46)	31.67 (3.71)																	
***DST% Variability (CV)***					ANOVA	0.0024	0.0187	0.321	0.336	0.646	0.204	0.524	0.0471	0.017	0.416	0.254	0.997	0.794	0.779	0.852	
					ANCOVA	0.0118	0.327	0.317	0.488	0.506	0.539	0.812	0.039	0.0048	0.769	0.336	0.818	0.75	0.152	0.384	0.606
Freezers	7.03 (4.04)	10.11 (9.17)	8.92 (5.72)	8.27 (6.47)																	
non-Freezers	5.07 (2.62)	4.96 (3.82)	4.24 (1.74)	4.65 (3.00)																	
***Velocity (cm/sec)***					ANOVA	0.0004	<0.0001	0.154	0.274	0.0142	<0.0001	0.596	<0.0001	0.815	0.0054	0.31	0.605	0.708	0.0007	0.904	
					ANCOVA	0.07	0.983	0.867	0.965	0.0467	0.0016	0.173	0.0487	0.9968	0.0064	0.657	0.269	0.941	0.403	0.957	0.069
Freezers	88.26 (26.52)	84.02 (29.12)	74.83 (26.50)	78.26 (29.27)																	
non-Freezers	119.90 (20.43)	109.51 (16.02)	111.16 (17.19)	112.43 (12.39)																	

DT, Dual-Task; VC, Visual Cues; UPDRS-III, Unified Parkinson’s Disease Rating Scale-III; DST%, Double Support Time Percentage

* illustrates interactions between groups, conditions, and trials (referring to the statistical comparisons between subsequent trials to evaluate whether completing each condition three times influences behaviours found).

**Fig 3:**
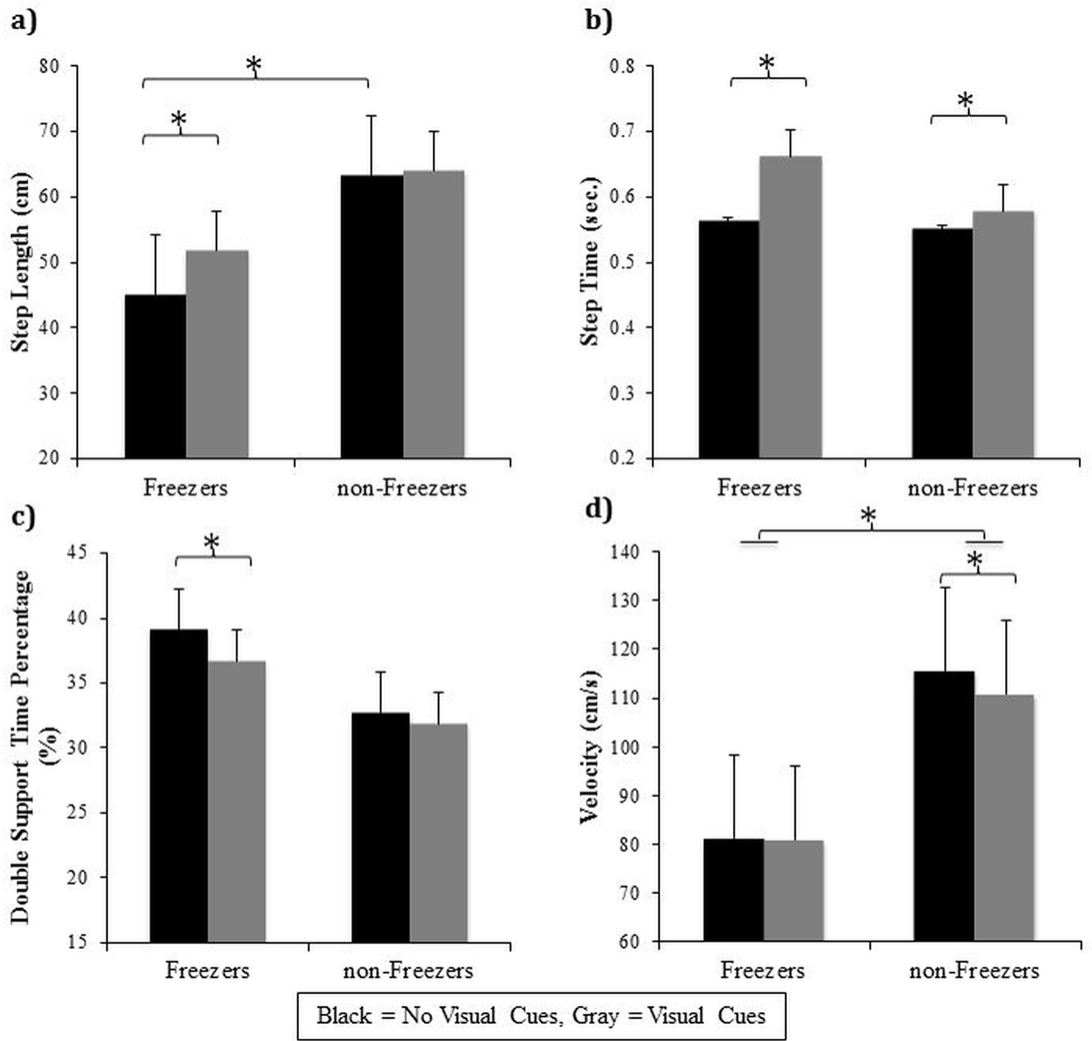
Significant gait interactions in Experiment #1 between group and visual Cues: Graphical illustration of a) step length, b) step time, c) double support time percentage, and d) velocity of the freezers and non-freezers as a result of walking toward and through a narrowed doorway on either the “no visual cues” pathway (black) or the “visual cues” pathway (gray). Each parameter includes both “no dual-task” and “dual-task” conditions collapsed. * represents a significant difference at the p<0.05 level.

Significant interactions between group and visual cues were found for step length (F(1,29) = 10.91, p = 0.003), step time (F(1,29) = 6.86, p = 0.014), DST% (F(1,29) = 5.21, p = 0.03), and velocity (F(1,29) = 6.80, p = 0.014) (see Fig 3). Overall, freezers walked with a significantly smaller step length (p<0.001), and with a reduced velocity (p<0.001) compared to non-freezers in the absence of visual cues. Furthermore, Tukey’s post-hoc revealed that only the freezer group significantly increased their step length (p<0.001) and reduced their DST% (p = 0.001) when visual cues were available compared to no visual cues conditions. Additionally, both freezers and non-freezers significantly increased their step time (p<0.01) when visual cues were provided. However, only the non-freezer group significantly reduced their velocity (p = 0.027) when visual cues were provided compared to no visual cues conditions. When severity was controlled for (ANCOVA), the significant interactions for step length, step time, DST%, and velocity found between group and visual cues remained significant.

**Fig 4:**
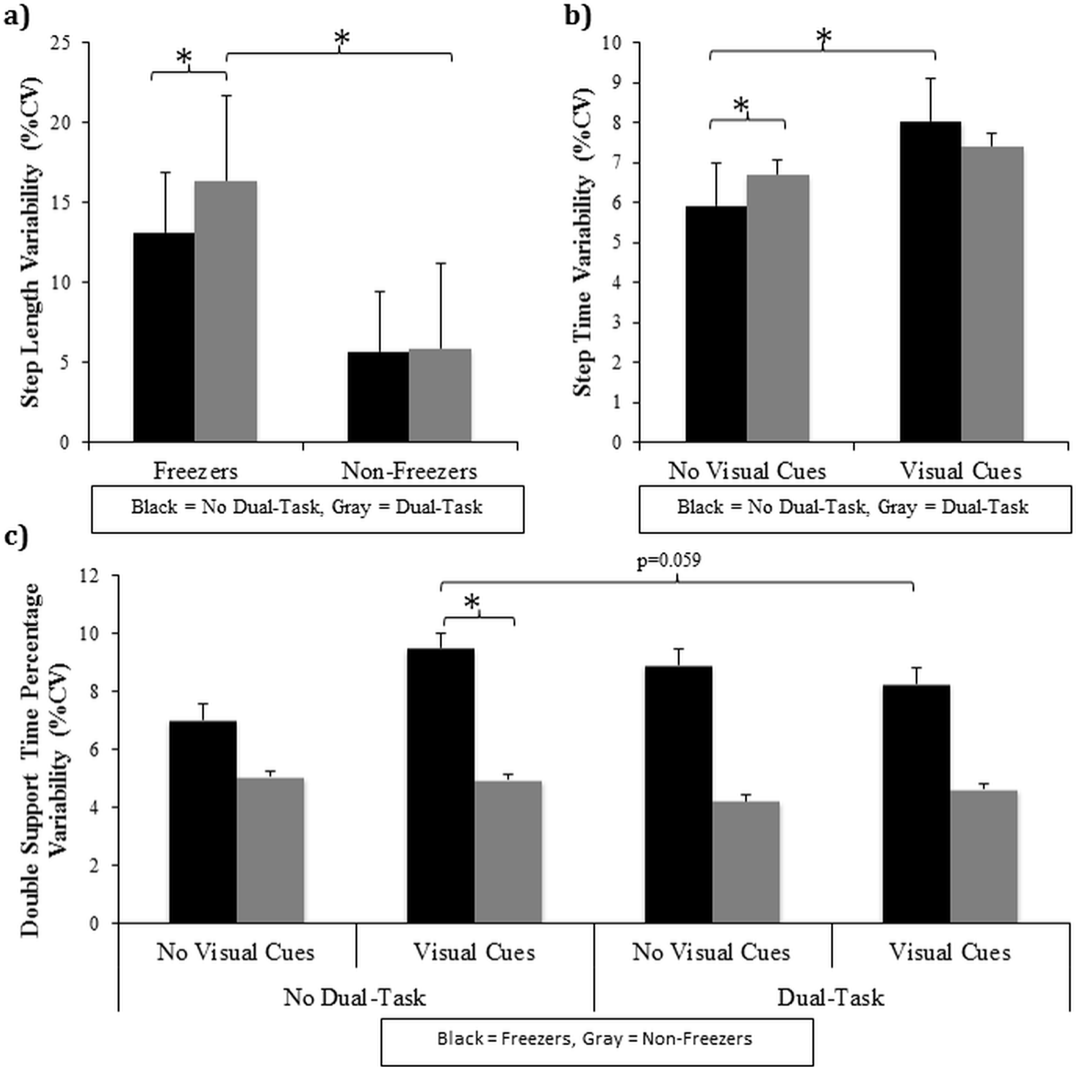
Significant gait variability interactions in Experiment #1: Graphical illustration of a) group x dual-task interaction for step length variability, b) dual-Task x visual cues interaction for step time variability, and c) group x dual-task x visual cues interaction for double support time percentage variability. * represents a significant difference at the p<0.05 level.

A significant interaction between group and dual-task was found for step length variability (F(1,28) = 6.67, p = 0.015)(see Fig 4). Tukey’s post hoc analysis revealed that only the freezers significantly increased step length variability when the dual-task was performed (p = 0.001) compared to walking without a dual-task (and compared to non-freezers, p = 0.028). When severity was controlled for, this significant interaction between group and condition approached significance (F(1,27) = 3.84, p = 0.061).

On average, freezers walked with a significantly greater step time variability than non-freezers illustrated by a main effect of group (F(1,29) = 7.16, p = 0.012). Of greater interest, a significant interaction between dual-task and visual cues was found for step time variability (F(1,29) = 12.05, p = 0.002)(see Fig 4). Tukey’s post hoc analysis demonstrated firstly that all PD participants had significantly greater step time variability when walking while performing a dual-task (p = 0.035) compared to no dual-task (+DT/-VC vs.–DT/-VC). Secondly, all PD participants also had greater step time variability when visual cues were present while walking (p<0.001) compared to no visual cues (-DT/+VC vs.–DT/-VC). Finally, when visual cues were available, step time variability did not change, in either PD group, when asked to simultaneously perform a dual-task compared to no dual-task (+DT/+VC vs.–DT/+VC). A significant interaction between condition and trial (F(2,58) = 4.04, p = 0.023) revealed that all participants had significantly greater step time variability on the first trial compared to the subsequent trials (p<0.049) but only in the absence of a dual-task. When controlling for severity, the significant main effect described above for step time variability was no longer significant.

Of greatest interest, a statistically significant interaction between group, dual-task, and visual cues was found for DST% variability (F(1,27) = 6.45, p = 0.017)(see Fig 4). Tukey’s post hoc analyses revealed freezers had significantly greater DST% variability (p = 0.03) compared to non-freezers, specifically when they walked with visual cues available but in the absence of a dual-task (-DT/+VC). Additionally, there was a near significant decrease in DST% variability within the freezer group (p = 0.059) specifically when the dual-task was combined with visual cues (+DT/+VC vs–DT/+VC). When controlling for severity, this interaction between group, dual-task and visual cues remained significant.

#### Gaze results

**Fig 5:**
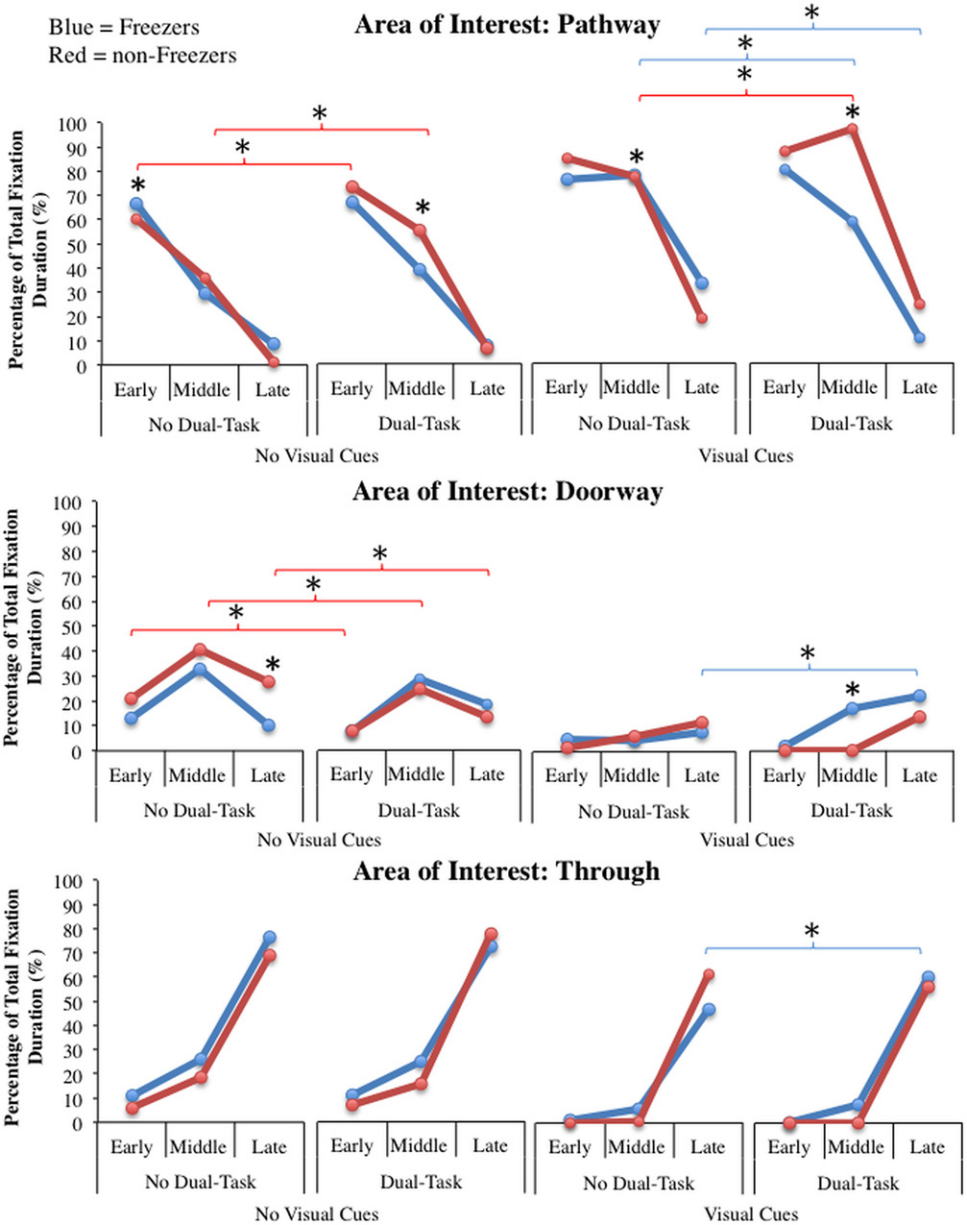
Significant gaze interactions in Experiment #1 between group, dual-task, visual cues, phase, and area of interest: Graphical illustration of the percentage of total fixation duration towards the pathway (top graphs), doorway (middle graphs) and through the doorway (bottom graphs) of the freezers (blue) and non-freezers (red) in the early, middle and late phases of walking toward and through the narrowed doorway in the “dual-task” or “no dual-task” conditions on either the “no visual cues” pathway or the “visual cues” pathway. * represents a significant difference at the p<0.05 level uncovered with Fisher’s LSD analysis. Blue parenthetical lines represent differences within the freezer group, and red parenthetical lines represent differences within the non-freezer group.

Significant five-way interactions between group, dual-task, visual cues, phase, and area of interest were found for percentage of total fixation duration (F(4,108) = 2.99, p = 0.022; see Fig 5) and percentage of fixations (i.e. frequency of gaze) (F(4,108) = 3.18, p = 0.016). Tukey’s HSD analysis revealed few significant patterns of gaze behaviour, whereas Fisher’s LSD uncovered numerous key differences.

At baseline, Tukey’s HSD did not reveal any key differences. In contrast, Fisher’s LSD uncovered that at baseline, (-DT-VC), freezers looked toward the pathway significantly longer (p = 0.044) and more frequently (p = 0.021) (and thus less toward the doorway, p = 0.072) than non-freezers in the early phase. During the middle phase, there were no differences in gaze behavior between groups. However, during the late phase, freezers looked toward the doorway significantly less (p = 0.018) and for a shorter duration (p = 0.009) compared to non-freezers.

When a dual-task was added (+DT/-VC), Fisher’s LSD (but not Tukey’s) revealed that only the non-freezers looked more toward the pathway and for a longer duration (thus, looked less toward the door, p<0.007) compared to no dual-task (-DT/-VC) in the early (p = 0.019) and middle phases (p = 0.001). Notably, non-freezers look toward the pathway more than the freezers when performing a dual-task (+DT/-VC) specifically in the middle phase (p<0.04). Additionally, non-freezers reduced the frequency (p = 0.066) and duration (p = 0.033) of gaze toward the doorway when a dual-task was present compared to baseline (+DT/-VC vs.–DT/-VC), specifically in the late phase.

Alternatively, Fisher’s LSD demonstrated that when visual cues were added, all participants looked significantly more toward the pathway compared to the other areas of interest (i.e. at the doorway and through the doorway), during the early and middle phases. However, in the late phase, freezers looked to the pathway significantly longer (p = 0.011) and with greater frequency (p = 0.012) than non-freezers. Tukey’s HSD did not reveal these key differences.

Lastly, Fisher’s LSD showed that when the dual-task was combined with visual cues (+DT/+VC), freezers significantly decreased the duration (p = 0.003) and frequency (p = 0.005) that they looked towards the pathway (compared to the -DT+VC condition), whereas non-freezers significantly increased the duration (p<0.001) and frequency (p = 0.001) that they looked towards the pathway, only in the middle phase. Likewise, freezers increase gaze duration (whereas non-freezers decreased gaze duration) towards the doorway (compared to–DT/+VC condition). In the late phase, only the freezer group increased their gaze (frequency and duration) toward the doorway (p = 0.023) and through (p = 0.03) the doorway, while looking less at the pathway (p<0.001) (compared to–DT/+VC). The only key finding that Tukey’s HSD analysis demonstrated was that non-freezers looked toward the pathway significantly longer (p = 0.002) and more frequently (p = 0.001) than freezers in the middle phase when the dual-task was combined with visual cues (+DT/+VC).

#### Skin conductance

A significant interaction between dual-task and visual cues was found for skin conductance (F(1,37) = 30.93, p<0.001). Tukey’s post hoc analysis revealed that all PD participants had significantly higher skin conductance levels (p = 0.032) when walking while performing a dual-task compared to no dual-task (+DT/-VC vs.–DT/-VC). When visual cues were available, all participants had significantly lower skin conductance level (p = 0.015) compared to walking without visual cues (-DT/+VC vs.–DT/-VC). Interestingly, when the dual-task was combined with visual cues, all participants demonstrated significantly higher skin conductance levels compared to walking with a dual-task but without visual cues (p<0.001) (+DT/-VC) and compared to walking with visual cues but without a dual-task (p<0.001) (-DT/+VC). Notably, there were no differences in skin conductance between freezers and non-freezers, even at resting baseline.

#### Dual-task errors

There were no significant results for dual-task errors.

### Discussion

#### Freezing of gait: Is it a cognitive problem?

The first aim of experiment #1 was to understand whether diverting attention with a dual-task influences FOG. As expected, the current study found that freezers demonstrated a greater number of FOG episodes and percentage of trial spent frozen when performing the dual-task compared to baseline (when visual cues were not provided). The dual-task also resulted in decreased step length and velocity and increased step time variability and DST% variability in both the freezers and non-freezers compared to baseline. Importantly, the dual-task only increased step length variability in the freezers, but not the non-freezers. This was an important finding since step length variability has often been argued to predict an upcoming FOG episode [2,3,5,8,15,39]. Overall, these findings support previous work that contribute to the cognitive model of FOG [10–12,40–44], which suggests that allocating attention away from walking, or increasing the demands on attention, may be responsible for freezing episodes.

The second aim of experiment #1 was to address whether performing a dual-task interferes with one’s ability to use visual cues to improve gait. If visual cues improve gait through an attentional strategy, then diverting attention with a dual-task was expected to reduce freezers’ ability to use visual cues, thus impairing gait. However, visual cues significantly improved step length, step time, and DST% in freezers, regardless of whether the dual-task was performed or not (-DT/+VC and +DT/+VC). Notably, since dual-task performance (+DT/+VC) matched that of baseline (in which prioritization was given to only counting numbers), we conclude that prioritization of attention was given to the secondary task and not foot placement on the visual cues. To accomplish the secondary task correctly, participants were required to continually process previously retrieved information throughout the trial while monitoring for further designated numbers. Therefore, correct performance on the dual-task suggests that participants prioritized the secondary task throughout the entire trial. Although, if decrements to dual-task performance were found, it could not have been confirmed where (throughout the trial) a change in prioritization of the secondary task took place. This is an inherit limitation of the secondary task utilized which can be further explored in the future. The presence of visual cues also reduced the occurrence of FOG episodes. However, contradicting the spatiotemporal gait parameters, in the few freezing episodes that were captured, visual cues were not able to ameliorate FOG while the dual-task was performed, suggesting that the dual-task interfered with the freezers’ ability to utilize the visual cues. This would support the notion that visual cues ameliorate FOG through an attentional strategy, and diverting attention away from gait, therefore, underlies these debilitating FOG episodes. The contradiction between these findings is somewhat puzzling. Since only four participants experienced freezing episodes and the severity of freezing did not match the gait parameters, cautious interpretations of the FOG measurements were made. Nevertheless, freezers walked with a significantly greater DST% variability compared to non-freezers when visual cues were present and no dual task was performed (-DT/+VC). It has been argued that increasing the variability in percentage of time spent in double support while walking towards or on a target is indicative of increased sampling of proprioceptive information [26]. Therefore, the visual cues may have directed greater conscious control of gait and sampling of proprioceptive information. For this reason, it was expected that the dual-task would disallow this conscious control of gait and influence an increase in sampling of proprioception, resulting in even greater variability in DST%. However, when the dual-task was performed while walking on the visual cues, freezers’ variability in DST% decreased almost significantly (p = 0.059), and was similar to non-freezers. Therefore, visual cues may allow freezers to continue utilizing proprioception and performance of the dual-task without blocks in motor output. In support of these findings, step time variability in both groups increased when visual cues were available to step on without simultaneous performance of the dual-task. With the dual-task, step time variability did not increase when visual cues were present; this variability was less than the variability associated with walking on the visual cues without the dual-task, although not statistically significant. These gait findings begin to suggest that an attentional mechanism does not entirely underlie FOG, which is further supported by evidence from the gaze behavior findings.

It was expected that if an attentional impairment is responsible for the mechanism underlying FOG, then increasing demand on attention might demonstrate different patterns of gaze behaviour in freezers and non-freezers. For this reason, we aimed to determine whether freezers visually sample narrow spaces differently than non-freezers and how a dual-task may influence this behaviour. This study found that at baseline (no dual-task) in the early phase while approaching a narrow doorway, freezers looked to the pathway more than non-freezers. In the late phase, freezers looked to the doorway significantly less than non-freezers. Therefore, those who experience FOG may have adopted a gaze behaviour strategy aimed to decrease the perceived threat of the upcoming doorway, thereby decreasing demand on attentional resources that are loaded by conscious control of walking [45]. Interestingly, when the dual-task was performed and attentional load was increased (-DT/-VC vs. +DT/-VC), freezers did not change their gaze sampling behaviour compared to baseline. However, non-freezers’ gaze increased towards the pathway and decreased towards the doorway when performing the dual-task. Therefore, the dual-task influenced non-freezers to adopt a gaze strategy similar to freezers. Performance of the digit-monitoring task also significantly increased skin conductance levels in both groups, suggesting that heightened arousal might have led to greater attentiveness to the tasks that included the dual-task, but resulted in more impaired gait. If FOG is the result of an attentional deficit, freezers may have adopted a compensatory strategy in an attempt to minimize arousal associated with walking (looking less to the doorway) to minimize demand on attentional resources. Performance of a dual-task that further increased demand on attention would therefore not be expected to change gaze behaviour in freezers since they had already employed a strategy to minimize arousal without the dual-task. This is supported by the findings where non-freezers adopted the same strategy as freezers when performing the dual-task that increased arousal. Therefore, freezers may be compensating for increased demand on attention with visual behaviour, supporting the cognitive model of FOG.

When the visual cues pathway was provided and the dual-task was not performed, freezers and non-freezers behaved similarly, fixating longer and more frequently towards the pathway in the early and middle phases, while looking more through the doorway in the late phase. In addition to greater fixations towards the pathway, improved gait, and the suggested heightened use of proprioception (DST% variability increase), arousal significantly decreased when visual cues were provided (no dual-task) compared to baseline. Decreased arousal might suggest that although freezers and non-freezers are looking towards the visual cues on the pathway, visual cues may be improving gait through a sensory mechanism.

Remarkably, freezers decreased the frequency and duration of gaze towards the pathway and increased visual sampling of the doorway (potential threat) (middle and late phase), whereas the non-freezers increased visual gaze towards the pathway (middle phase) while performing the dual-task with visual cues present (+DT/+VC). This might suggest that with increased attentional demand, visual cues fostered improved planning for the doorway in freezers. Therefore, visual cues may have reduced processing demands to allow sampling of the threatening doorway, which freezers avoided without visual cues. If planning is a key contributor to FOG, than further sampling of the doorway may resolve the mechanism underlying freezing when there is greater demand on processing resources. Since non-freezers might possess greater planning efficiency, further sampling of the doorway may not have been necessary. Performing the dual-task with visual cues to walk on also significantly increased arousal compared to performing the dual-task without visual cues. Previous research has suggested that heightened arousal may increase attention and vigilance [29–31], and has even been shown to facilitate sensory processing [46,47]. Therefore, these findings together might suggest that when arousal levels were elevated (i.e. in the +DT/+VC condition) participants may have been able to improve processing of visual cues, and subsequently gait impairments were overcome.

If increased demand on attention is the underlying mechanism of FOG (cognitive model), then visual cues should not have improved gait while performing a dual-task, or permitted increased visual sampling of potentially perceived threats to gait. Therefore, the current study suggests that FOG is not exclusively the result of allocating attention away from gait or increasing attentional demand, but perhaps visual cues provided a sensory strategy to compensate for impaired proprioception that may strongly influence FOG (sensory-perceptual model) [25–27]. To examine the sensory-perceptual influence on FOG, sensory feedback was restricted by removing vision of the participants’ lower-limbs in the second experiment.

## Experiment 2: The influence of sensory feedback on freezing of gait

### Methods

Three additional conditions were added to examine the influence of restricting sensory feedback on FOG: (i) Lower-limb occlusion (LLO)–walking through the narrow doorway without vision of the lower-limbs; (ii) Lower-limb occlusion and visual cues (LLO+VC)–walking through the narrow doorway with vision of the lower-limbs occluded and the aid of visual cues; (iii) Lower-limb occlusion, visual cues, and dual-task (LLO+VC+DT)–walking through the narrow doorway with vision of the lower-limbs occluded and the aid of visual cues while performing the dual-task. There were three trials performed for each condition and starting procedures were the same as previously described in the general methods.

### Data Analysis

#### Participant demographic analysis

In order to verify if there were significant differences between the freezers and non-freezers, independent t-tests were utilized.

#### Gait data and statistical analysis

In the second experiment, data was collected from two independent groups: 15 freezers and 20 non-freezer participants. All freezing of gait and spatiotemporal gait parameters used the same techniques and were analyzed identically to experiment#1 (Table 4 presents the number of FOG episodes and average percentage of trial spent frozen for each patient whom experienced freezing in the present study). A three factor mixed repeated measures ANOVA (group x condition x trial) assessed group differences in each condition. Again, UPDRS-III scores were included as a covariate variable on a secondary analysis (ANCOVA). Tukey’s Honest Significant Difference post hoc was utilized to determine where significant differences were with respect to main effects and interactions.

**Table 4:** Number of freezing of gait episodes and average percentage of trials spent frozen with respect to the four individuals with Parkinson’s disease who experienced freezing of gait episodes in both experiments collapsed.

Patient	Number of FOG Episodes					Average Percentage of Trial Spent Frozen (%)				
	No Dual-Task			Dual-Task		No Dual-Task			Dual-Task	
	*No VC*	*VC*	*VC+ LLO*	*No VC*	*VC*	*No VC*	*VC*	*VC+ LLO*	*No VC*	*VC*
1	0	0	1	3	2	0	0	0.75	56.7	35.1
2	3	0	6	7	5	54.8	0	27.2	65.6	48.0
3	0	0	1	0	2	0	0	5.4	0	12.0
4	0	0	0	0	1	0	0	0	0	17.1

FOG, freezing of gait; VC, visual cues; VC+LLO, visual cues and lower-limbs occlusion

#### Gaze data and statistical analysis

The same methodology utilized in experiment#1 to investigate gaze behaviour was also employed in experiment#2. Participant’s gaze data was excluded from analysis if the displayed eye track was missing for more than 25% of the travel time [33]. For this reason, gaze data from 11 freezers and 17 non-freezers was reported on in experiment two. A four factor mixed repeated measures ANOVA (group x condition x phase x areas of interest) assessed group differences in each condition, and the three trials per condition were averaged. Similar to experiment #1, Tukey’s HSD post hoc test was utilized to determine where significant differences were with respect to main effects and interactions. Although, since no studies to date have investigated gaze behaviour in freezers while they navigate towards and through a doorway, and this study intends to guide future gaze behaviour research with regards to freezing of gait, Fisher’s LSD was also utilized and reported clearly in the results section.

#### Skin conductance data and statistical analysis

The same methodology utilized in experiment #1 to investigate electrodermal activity with Q sensor cuffs was conducted in experiment #2. A three factor mixed repeated measures ANOVA (group x condition x trial) assessed group differences in each condition. Tukey’s Honest Significant Difference post hoc was utilized to determine where significant differences were with respect to main effects and interactions.

### Results

#### Participant demographics

Data analysis did not uncover significant differences between groups with respect to age, shoulder width, visual distance acuity, or performance on the Montreal Cognitive Assessment. A significant difference was found between groups with respect to severity. The freezers (37.6) had a significantly higher UPDRS-III score than the non-freezers (24.7) (t(33) = 4.38, p<0.001, Table 5).

**Table 5:** Demographic characteristics of participants with Parkinson’s disease that experience freezing of gait (freezers) and do not experience freezing of gait (non-freezers) for individuals who participated in the second experiment.

Group	AGE	SEX	UPDRS-III	MOCA	Distance Acuity	Shoulder Width (cm)	TMT A (sec)	TMT B (sec)	DMT Forward (score/16)	DMT Backward (score/14)
Freezers (n = 15)	70.5 (7.23)	2 F, 13 M	37.6* (10.0)	25.1 (3.4)	20/50	47 (4.0)	51.5 (15.6)	154.8* (64.2)	10.6 (2.5)	6.1 (1.4)
Non-Freezers (n = 20)	70 (8.4)	2 F, 18 M	24.7 (7.5)	26.5 (2.1)	20/70	48.5 (4.3)	41.9 (12.8)	85.8 (34)	10.4 (2.7)	6.9 (1.8)

n, number of participants; UPDRS-III, Unified Parkinson’s Disease Rating Scale-III; MOCA

Montreal Cognitive Assessment; TMT A and B, Trail Making Test Parts A and B; DMT; Digit Memory Test.

* represents a significant difference between groups at the p<0.05 level.

#### Gait parameters

**Fig 6:**
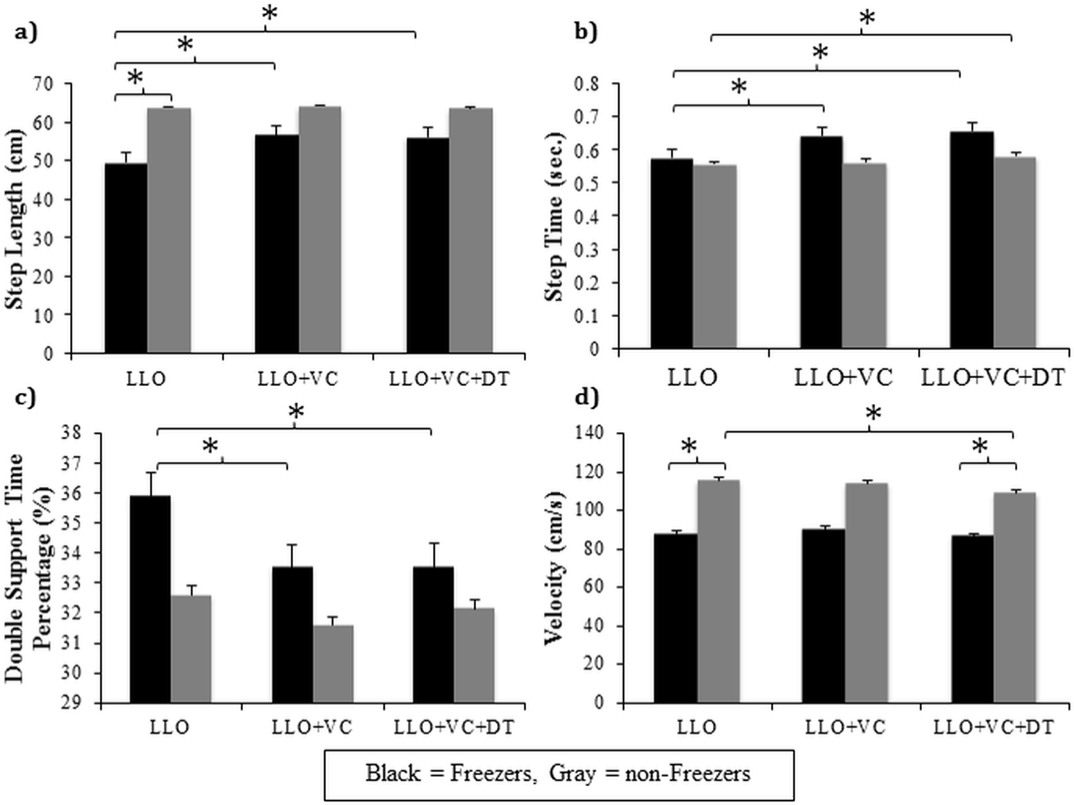
Significant gait interactions in Experiment #2 between group and condition. Graphical illustration of a) step length, b) step time, c) double support time percentage, and d) velocity of the freezers (black) and non-freezers (gray) as a result of walking toward and through a narrowed doorway in either the i) lower-limb occlusion (LLO), ii) lower-limb occlusion and visual cues (LLO+VC), or iii) lower-limb occlusion, visual cues and dual-task (LLO+VC+DT) conditions. * represents a significant difference at the p<0.05 level.

All significant and non-significant gait main effects and interactions are presented in Table 6. Significant interactions between group and condition were found for step length (F(2,55) = 11.17, p<0.001), step time (F(2,56) = 5.96, p = 0.005), DST% (F(2,56) = 5.07, p = 0.01), and velocity (F(2,56) = 3.17, p = 0.05). Tukey’s post hoc analysis revealed multiple key differences that can be found in Fig 6. Firstly, freezers walked with a significantly shorter step length (p<0.001) and slower velocity (p = 0.003) compared to non-freezers, specifically when the lower limbs were occluded (LLO). Freezers demonstrated significantly increased step length and step time, and a reduced DST%, during walking when visual cues were available (both with and without a dual-task: LLO+VC and LLO+VC+DT) compared to simply walking without vision of their lower limbs (LLO). Non-freezers also demonstrated a significant increase in step time, but a significant reduction in velocity specifically when walking with a dual-task in combination with visual cues and lower limb occlusion (LLO+VC+DT) compared to the LLO condition. Finally, freezers walked significantly slower than non-freezers (p = 0.037), specifically in the LLO+VC+DT condition. When controlling for severity, the significant interactions determined between condition and group for step length, step time, DST%, and velocity remained and the key differences previously discussed were identified by post hoc analysis.

**Table 6:** Experiment #2 spatiotemporal gait parameter effects and interactions.

	Conditions			Effects (p values)								
	LLO	LLO+VC	LLO+VC+DT		Group	Condition	Group* Condition	Trial	Group* Trial	Condition* Trial	Group* Condition* Trial	UPDRS
***Step Length (cm)***				ANOVA	0.0006	0.0002	<0.0001	<0.0001	0.42	<0.0001	0.216	
				ANCOVA	0.0036	0.103	0.0002	0.999	0.698	0.803	0.319	0.921
Freezers	49.77 (11.87)	56.78 (14.92)	56.35 (13.85)									
non-Freezers	63.88 (8.66)	64.33 (4.46)	63.73 (4.45)									
***Step Length Variability (CV)***				ANOVA	0.0145	0.0383	0.233	0.379	0.674	<0.0001	0.206	
				ANCOVA	0.131	0.917	0.435	0.992	0.858	0.969	0.793	0.295
Freezers	12.19 (9.77)	10.18 (9.16)	11.88 (10.10)									
non-Freezers	6.22 (3.82)	5.62 (2.38)	6.60 (3.54)									
***Step Time (sec.)***				ANOVA	0.0358	<0.0001	0.0045	<0.0001	0.629	0.109	0.313	
				ANCOVA	0.237	0.118	0.0222	0.243	0.536	0.21	0.305	0.281
Freezers	0.58 (0.10)	0.64 (0.14)	0.66 (0.11)									
non-Freezers	0.56 (0.06)	0.56 (0.06)	0.58 (0.06)									
***Step Time Variability (CV)***				ANOVA	0.07	0.0002	0.836	0.0304	0.057	0.0319	0.363	
				ANCOVA	0.541	0.648	0.914	0.707	0.156	0.693	0.773	0.09
Freezers	6.04 (3.47)	8.66 (5.43)	7.45 (3.45)									
non-Freezers	4.54 (2.07)	5.40 (1.96)	5.63 (2.03)									
***Double Support Time Percentage (%)***				ANOVA	0.868	0.0001	0.0095	0.497	0.678	0.0102	0.855	
				ANCOVA	0.332	0.087	0.0091	0.689	0.799	0.948	0.926	0.145
Freezers	35.90 (7.63)	33.51 (9.91)	33.55 (9.19)									
non-Freezers	32.62 (5.42)	31.59 (4.18)	32.15 (4.57)									
***DST% Variability (CV)***				ANOVA	0.053	0.354	0.974	0.625	0.553	0.6	0.411	
				ANCOVA	0.228	0.368	0.559	0.386	0.792	0.544	0.34	0.462
Freezers	6.57 (3.85)	6.22 (3.68)	6.83 (4.46)									
non-Freezers	5.06 (1.94)	4.80 (2.97)	5.46 (3.45)									
***Velocity (cm/sec)***				ANOVA	0.0014	0.0014	0.0496	<0.0001	0.635	<0.0001	0.083	
				ANCOVA	0.02	0.424	0.04	0.47	0.512	0.174	0.082	0.462
Freezers	88.12 (25.43)	90.53 (28.09)	86.73 (24.36)									
non-Freezers	115.39 (21.31)	113.96 (15.46)	109.04 (14.95)									

LLO, Lower-limbs occluded; LLO+VC, Lower-limbs occluded while walking on visual cues; LLO+VC+DT; Lower-limbs occluded while walking on visual cues and performing the dual-task; UPDRS-III, Unified Parkinson’s Disease Rating Scale-III;

* illustrates interactions between groups, conditions, and trials.

A main effect of group revealed that on average, freezers walked with a significantly greater step length variability than non-freezers (F(1,28) = 6.79, p = 0.015) and a greater DST% variability than non-freezers that approached significance (F(1,28) = 4.08, p = 0.053). A significant interaction between conditions and trial was observed for step length variability (F(4,112) = 7.78, p<0.001). Post hoc analysis revealed significant reductions in step length variability as the trials progressed specifically when walking with the lower limbs were occluded (LLO). When severity was controlled for, all main effects and interactions previously discussed for gait variability were no longer present.

#### Gaze results

**Fig 7:**
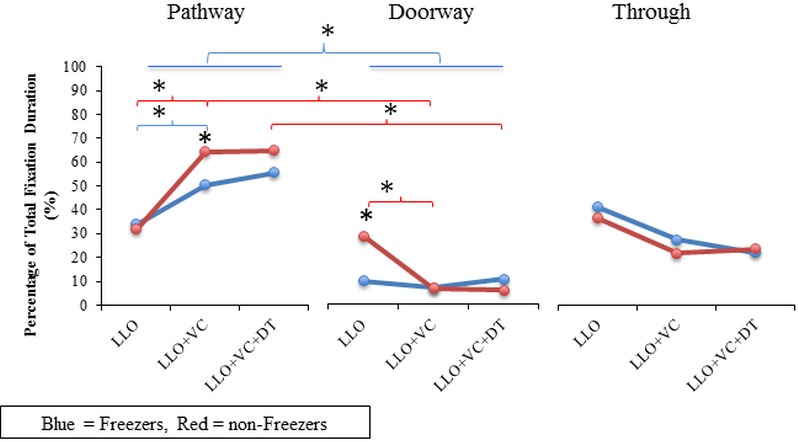
Significant gaze interactions in Experiment #2 between group and condition. Graphical illustration of the percentage of total fixation duration towards the pathway (left graph), doorway (middle graph) and through the doorway (right graph) of the freezers (blue) and non-freezers (red) in the i) Lower-limb occlusion (LLO), ii) Lower-limb occlusion and visual cues (LLO+VC), and iii) Lower-limb occlusion, visual cues and dual-task (LLO+VC+DT) conditions. * represents a significant difference at the p<0.05 level uncovered with Fisher’s LSD analysis. Blue parenthetical lines represent differences within the freezer group, and red parenthetical lines represent differences within the non-freezer group.

A significant interaction between group, condition, and area of interest was found for percentage of total fixation duration (F(4,96) = 2.49, p = 0.048) and multiple key differences were revealed by both the Tukey’s HSD and Fisher’s LSD post hoc analysis. The Fisher’s LSD differences are illustrated in Fig 7. Firstly, Fisher’s LSD demonstrated that freezers have significantly shorter gaze durations toward the doorway (compared to non-freezers) when walking with their lower limbs occluded (LLO condition) (p = 0.006). Additionally, freezers also look longer at the pathway compared to the doorway, whereas non-freezers split their gaze durations evenly across the different areas of interest. When visual cues were provided (LLO+VC and LLO+VC+DT), all PD participants significantly increased the duration in which they looked towards the pathway compared to LLO (regardless of dual-task) (p<0.018). Non-freezers looked at the pathway for significantly longer durations than freezers (p = 0.04) (and reduced gaze durations on the doorway) when visual cues were present in the absence of a dual-task (LLO+VC vs. LLO). With more conservative investigation, Tukey’s HSD confirmed that when visual cues were provided (LLO+VC and LLO+VC+DT), non-freezers significantly increased the duration in which they looked towards the pathway compared to LLO (p<0.001), whereas freezers did not. Tukey’s HSD also revealed that when visual cues were present, both freezers and non-freezers looked at the pathway significantly longer than the doorway (p<0.001).

#### Skin conductance

A significant main effect of condition (F(2,66) = 4.79, p = 0.011) was found for skin conductance levels. Tukey’s post hoc analysis revealed that all participants demonstrated significantly increased skin conductance while walking with a dual-task in combination with visual cues (LLO+VC+DT) compared to the LLO condition (p = 0.012). Notably, visual cues (LLO+VC) did not significantly change skin conductance levels compared to the LLO condition.

#### Dual-task errors

There were no significant results for dual-task errors.

### Discussion

#### Freezing of gait: Is it a sensory problem?

The first aim of experiment #2 was to investigate whether sensory deficits influence FOG. When vision of the lower-limbs was removed, step length and gait velocity were significantly worse in the freezers compared to non-freezers, and a greater amount of freezing episodes were observed compared to baseline. This suggests that the restriction of visual feedback about walking may have increased reliance on impaired proprioceptive processing, leading to more impaired gait in the freezers. In support of this, freezers generally walked with significantly greater step length variability and DST% variability (p = 0.059), suggesting greater sampling of proprioceptive behaviour [26]. Furthermore, at baseline (LLO), freezers directed gaze towards the doorway significantly less than non-freezers. Therefore, despite fixation duration predominantly towards the travel path observed in freezers, restricted sensory feedback promoted reliance on impaired proprioception that may have elicited a detrimental drive from the sensorimotor domain into the striatum, interfering with the original motor plan and causing decrements to gait [15], supporting the sensory-perceptual model of FOG. Since skin conductance levels during this condition (LLO) were minimal, cognitive and limbic involvements were most likely less responsible for the resulting gait impairment in this case.

The second experiment also aimed to investigate whether visual cues can improve freezing and gait impairments when sensory feedback was restricted. It was hypothesized that if visual cues compensate for proprioceptive deficits by visually guiding step placements, then removing vision of the lower-limbs may be expected to reduce the effectiveness of visual cues at improving gait. Contrary to our hypothesis, freezers walked with improved step length, step time, and DST% when visual cues were provided, as compared to the absence of visual cues. Since gait improved despite the inability to verify each step, visual cues may have either helped to focus attention on walking (cognitive model) or provided upcoming information which helped freezers form a feed forward plan, reducing the reliance on proprioceptive feedback (sensory-perceptual model). Gaze behaviour data demonstrated that visual cues directed freezers’ gaze towards the pathway (although not to the degree of non-freezers) to percentages significantly greater than the condition where visual cues were not present. This could be argued to indicate greater attention towards walking to improve gait (cognitive model), or reduced need for sampling of impaired proprioceptive information to improve gait (sensory-perceptual model), leaving the underlying mechanism unclear.

In order to fully understand the FOG mechanism that visual cues act on, the interaction between dual-task, restriction of sensory feedback, and visual cues was examined. While walking with a dual-task, visual cues, and restricted vision of the lower-limbs, freezers had significantly longer step length, greater step time, and decreased DST% compared to walking without visual cues or performing the dual-task (LLO+VC+DT vs. LLO). Therefore, since gait was improved despite increased attentional demand and restricted visual feedback from the lower-limbs, the visual cues may have provided upcoming information which helped freezers form a feed forward plan [13,17,32], a process found to be impaired specifically in those who experience FOG [17,18,48]. This is supported by the visual behaviour, in which freezers adopted a percentage of fixation duration towards the pathway similar to non-freezers, who may be able to form feed forward plans more effectively than freezers. On the other hand, visual cues may have provided rich optic flow to enhance feedback regarding self-motion from central and peripheral vision, which may relieve a sensory-perceptual deficit underlying FOG [49,50]. When investigating central vision, contributions of peripheral information cannot be ruled out, and may directly affect the mechanism underlying FOG. However, in order to elaborate on these aspects of gaze behaviour, future research could utilize virtual reality techniques to manipulate optic flow, and measure skin conductance levels to monitor arousal, so as to determine whether enriched self-motion feedback without heightened attention provides benefits. Interestingly, complimentary to the findings from experiment #1, when the dual-task was performed while patients walked on the visual cues without vision of their lower-limbs, skin conductance levels were significantly greater than restricted vision of the lower-limbs only (LLO vs. LLO+VC+DT), but gait was still improved by the visual cues. This further supports the notion that increased arousal might enhance task-relevant sensory processing (such as visual cues to foster feed-forward planning), and thus gait will benefit. However, visual cues did not improve freezing during a dual-task or when vision of the lower limbs was restricted. This might suggest that central vision is needed to compensate for proprioceptive deficits that make it difficult for freezers to assess their forward progression toward an obstacle as well as their actual step amplitude.

Since there were few FOG episodes elicited in only a small portion of the patients and the severity of freezing did not match the gait parameters, cautious interpretations of the FOG measurements were made. Visual cues helped improve gait regardless of dual-task or restricted vision of the lower-limbs, but were unable to reduce the amount of freezing episodes overall. These differences between gait data and FOG observations contrasts previous research [2,4,15] and therefore, inferences were made with greater emphasis on the gait parameters.

## General Discussion

### Freezing of gait: A cognitive and sensory overload problem

To our knowledge, this is the first study to comprehensively explore the interaction between the cognitive and sensory-perceptual influences on FOG in PD using gait, gaze, and skin conductance. Since attentional and sensory manipulations were found to influence gait in freezers, this study supports the notion that an overload of processing resources may consequentially block motor output, resulting in FOG [18,42–44,51]. Multiple domains (cognitive, sensorimotor, and limbic) project to the basal ganglia and converge at the level of the striatum [51]. It has been suggested that when domains that project to the basal ganglia require greater demand on striatal dopamine reserve (processing resources), FOG occurs [51]. In the present study, dual-tasking and restricting vision of the lower limbs may have increased the load on processing resources in freezers specifically, explaining worsened gait with these manipulations [44,51]. The gaze behaviour would support the argument that freezers may utilize vision to compensate for increased demand on processing resources. By minimizing gaze directed towards the doorway, freezers may have been attempting to decrease the demand on processing resources associated with the perceived threat of the doorway. Furthermore, this may be the reason why non-freezers adopted gaze behaviour similar to freezers when attentional load was increased (ie. dual-task). Visual cues may have aided freezers by decreasing the demand on processing resources required by gait through reliance on alternate neural pathways that perhaps involve the cerebellum [52–56]. When movements (such as walking) are guided externally (ie. visually guided locations for a footfall), cerebellar contributions to motor control increase, relieving demand on processing resources associated with the basal ganglia [52–55,57]. With less demand on these processing resources through cerebellum compensation, more resources may be available for projections from cognitive, sensorimotor, and limbic domains to the striatum [51]. In support of this, when visual cues were present, despite performing the dual-task, freezers were able to attend more to the relevant threat (doorway) while continuing to receive gait benefits from the visual cues. The observation that increased duration and frequency of gaze towards the visual cues (which improved gait even when attentional load was increased and sensory feedback was restricted) supports the suggestion that visual cues may decrease demand on processing resources by ameliorating freezers’ inability to effectively form a feed forward movement plan [17,18,48]. Future work should investigate the effect that dopaminergic medication has on the interaction between attentional and sensory-perceptual contributions to FOG in fMRI studies. Measures of anxiety associated with the interaction between attentional and sensory-perceptual contributions to FOG should also be further investigated since freezers increased gaze towards the threat to movement control (the doorway), perhaps to minimize its demand on processing resources. Likewise, whether specific fixation patterns (ie. looking to the doorway versus the pathway) can provide effective strategies to alleviate freezing requires future investigation.

## Conclusion

The results of the present study suggest that although diverting attention does elicit freezing episodes, attention is not exclusively responsible for freezing since visual cues were able to improve gait despite performing a dual-task. Moreover, gait was impaired in freezers when vision of the lower-limbs was removed, but improved when visual cues were provided despite the inability to verify each step. Visual cues were additionally able to improve gait when both a dual-task was performed and vision of the lower-limbs was removed. Therefore, it was suggested that FOG may be the result of an overload of processing resources, and visual cues may decrease the demand on processing resources by promoting gait control through alternative pathways.

## Supporting Information

S1 File Data underlying spatiotemporal gait parameter findings. Experiment #1 and #2.
(XLSX)

S2 File Data underlying gaze behaviour findings. Experiment #1 and #2.
(XLSX)

S3 File Data underlying skin conductance level findings. Experiment #1 and #2.
(XLSX)

S4 File Participant demographics and dual-task errors. Experiment #1 and #2.
(XLSX)
